# Telemedicine for Epilepsy Support in Resource-Poor Settings

**DOI:** 10.3389/fpubh.2014.00120

**Published:** 2014-08-21

**Authors:** Victor Patterson

**Affiliations:** ^1^Dhulikhel Hospital, Dhulikhel, Nepal

**Keywords:** epilepsy, untreated epilepsy, phone app, telemedicine, teleneurology, epilepsy treatment gap, developing countries

## Abstract

**The Problem:** Epilepsy is a common disease worldwide causing significant physical and social disability. It is one of the most treatable neurological diseases. Yet, in rural, poorer countries like much of India and Nepal, most people with epilepsy are not undergoing any treatment often because they cannot access doctors.

**Conventional Approaches:** It is being appreciated that perhaps doctors are not the solution and that enabling health workers to treat epilepsy may be better. Few details, however, have been put forward about how that might be achieved.

**Thinking Differently:** Untreated epilepsy should be considered a public health problem like HIV/AIDS, the various steps needed for treatment identified and solutions found.

**Telemedicine Approaches:** Telemedicine might contribute to two steps – diagnosis and review. A tool that enables non-doctors to diagnose episodes as epileptic has been developed as a mobile phone app and has good applicability, sensitivity, and specificity for the diagnosis. There are a number of ways in which the use of phone review or short messaging service can improve management.

**Conclusion:** Telemedicine, as part of a public health program, can potentially help the millions of people in the resource-poor world with untreated epilepsy.

Telemedicine is not an end in itself but a method of medical practice, which either enables the reach of medicine to be extended beyond what can be done by conventional means or enables medicine to be practised more efficiently or effectively than by conventional means (1). Too often telemedicine papers start with the technology and perhaps deal with the problem later. This paper will start with a detailed evaluation of the problem of untreated epilepsy, discuss the necessary strategies to solve it, and finish with how telemedicine might fit into those strategies in either of the roles above.

## Problem of Untreated Epilepsy

Epilepsy is a common disease worldwide. Its prevalence is approximately 1 in 200 people but most studies from the poorer parts of the world have shown a prevalence of about twice that (2). This increase has been attributed to a combination of brain infections such as neurocysticercosis, poorer obstetric care leading to more perinatal brain disease, and more head trauma.

The effects of uncontrolled epilepsy are potentially serious. Death in epilepsy is estimated to occur with increased frequency in both rich and poor countries. There are two main causes for this: (1) accidents, in particular falls and drowning, where someone has a seizure in a vulnerable position and (2) sudden unexplained death in epilepsy (SUDEP) in which having a seizure leads to death by unexplained but possibly multifactorial causes (3). Seizures occurring when the person is in vulnerable positions can also cause disabilities ranging from burns to fractures and brain injury. Its arguably much more common effect is the social isolation which it produces. This may be in the form of family concern for the person’s vulnerability and safety or by society’s view of the person’s disease. Societal prejudice against people with epilepsy is not new but in richer countries it has been lessened by public education emanating from patient-based charities and public health agencies. This problem is much greater in poorer societies where often epilepsy is not recognized as a medical condition at all and notions such as possession by evil spirits are more widespread. The stigma of epilepsy is a particular problem for girls with epilepsy as their marriage prospects are significantly diminished by this diagnosis (4).

Yet, epilepsy is a treatable condition. In resource-rich countries about two-thirds of people with it have their seizures completely controlled on simple medicines (5). This makes it one of the most treatable conditions not only just within neurology but also in medicine with a number needed to treat (NNT) to effect one cure of two. In rich countries, the treatment gap, the number of people not on treatment as a percentage of the total population with epilepsy, is negligible but in poorer countries it averages 75% in the many studies where it has been ascertained (2, 6). There are a number of reasons for this treatment gap: inability to pay for long-term medicines is one; lack of awareness that epilepsy is a medical disease and lack of doctors are others (6–8). The last is particularly prevalent in countries such as India and Nepal where most people live in rural areas and almost all doctors live in cities.

## Conventional Approaches

The International League against Epilepsy (ILAE) and the World Health Organization (WHO) jointly conceived a global campaign against epilepsy in 1997. This supported a number of pilot projects in a number of different countries but its efforts have had no generalizable effect on the problem throughout the resource-poor world and it has largely fizzled out (9). Since one of the problems is a large shortage of doctors in rural areas and since there has been no obvious solution to the scale of this problem, it is being increasingly appreciated that perhaps increasing doctors is not the solution and that enabling health workers to treat epilepsy may be the way forward (2, 8, 10). Few details, however, have been put forward about how this might be achieved. A detailed proposal about how untreated epilepsy might be dealt with in India using existing doctors has been published but has not been implemented (11).

## Thinking Differently – A Public Health Approach

Although epilepsy in general is regarded as a neurological problem, and therefore, within the remit of neurologists almost exclusively, it might be more useful to regard *untreated* epilepsy as a public health problem, and extend its remit much wider than just neurologists. This approach would actually deal with untreated epilepsy in the same way that malaria and HIV/AIDS are, even though they both are infectious diseases.

As part of this approach the individual steps for both community and individual management need to be defined and then ways of dealing with each of these steps determined, a type of project management. Once these steps are identified then solutions to them can be devised, tested, and hopefully funded. This is essentially the approach that has been used in HIV/AIDS.

## Defining the Steps

A summary of the possible components needed for a public health approach to untreated epilepsy is shown in Table 1.

**Table 1:** **Necessary steps for untreated epilepsy**.

**Community**	Prevention
	Awareness
**Individuals**	Identification
	Diagnosis
	Treatment
	Education
	Review

Each of these steps can improve matters to some extent in isolation; for example, identifying, diagnosing, and treating untreated epilepsy resulted in 50% of people being maintained on treatment at 8 months without any review arrangements (12). However, they are likely to be much more effective when combined.

If non-doctors, particularly village health workers are to play a role in managing UE then they need to be empowered to carry out these steps, and in particular, those which are traditionally done by doctors such as diagnosis, treatment, and review. In order to see whether telemedicine will contribute to untreated epilepsy management then these steps need to be broken down into smaller steps. For the diagnosis of epilepsy these would be: first, *Is the episode an epileptic seizure or not?*; second, *If it is, is it primary or secondary epilepsy?*; third, *Does it need treatment or not?*

The first step is the most important as the consequences of getting it wrong are significant – either people who do not have epilepsy are diagnosed as having epilepsy and put on unnecessary and ineffective medication or people with epilepsy who might benefit from these drugs are diagnosed as not having epilepsy and so deprived of them. Surprisingly, even in rich countries, this diagnosis is made entirely from a history of the episode and not by any examination or investigation – there is a common misconception that an electroencephalogram (EEG) is necessary at this stage. Diagnosis is essentially a problem of pattern recognition and the more experienced the doctor then the more likely the diagnosis is to be correct. Even in the best hands there is still a surprisingly high rate of misdiagnosis, at least in those patients reaching a tertiary referral center where estimates of 20% are widely accepted (13, 14). It is therefore not possible for non-doctors to take on this role unless they can be provided with a tool, which distils and applies the knowledge of an experienced epilepsy doctor.

The second step, whether the epilepsy is primary, usually genetic in origin, or secondary, due to some structural brain abnormality, is important in deciding which anti-epileptic drug to prescribe. Sometimes this is clear from the clinical history, but where there is uncertainty an EEG is useful. EEGs are not widely available so a clinical algorithm of some sort will be necessary if this task is to be devolved to non-doctors and work on this task is in progress.

The third step, whether the epilepsy needs treating, is easily dealt with by a simple algorithm.

For treatment, the options will depend on local circumstances and simple algorithms to choose and start available medications are easily provided.

Review is more complex and currently requires the interaction of an experienced doctor with the patient, conventionally face-to-face. The shortage of experienced doctors in rural areas makes this approach impossible. Either empowering non-doctor health workers to consult face-to-face or using technology to enable remote consultations are possible alternatives.

## Telemedicine in Untreated Epilepsy: A Useful Tool

The above analysis suggests that a conventional system of medical care based on face-to-face consultation with a doctor will not be able to diagnose or review people with untreated epilepsy because there are simply not enough doctors. There seems, therefore, to be two distinct ways in which telemedicine solutions can help: first, by devolving care to non-medical health workers and empowering them with appropriate tools so that they can diagnose and review appropriately; second, by providing some services remotely using a combination of medical and non-medical personnel. Each of these might be useful in different situations.

### Diagnosis

A diagnostic tool to determine whether episodes of loss of consciousness are due to epilepsy or to other causes has been developed (15). This is based on the answers to 11 selected questions and results in a probability score of the episodes being epilepsy. It is broadly based on a Bayesian analysis of the likelihood of symptoms being associated or not associated with epilepsy. The selected questions were those with the highest likelihood ratios (16) of either being epilepsy or not epilepsy.

The probability score derived by this has been validated first in a small population from Nepal and second from three larger populations, two in Nepal and one in India (Victor Patterson et al., submitted for publication). The way the algorithm has been devised reflects the Bayesian way in which experienced doctors make a diagnosis of epilepsy – starting with a pre-test probability and then asking a series of questions, which individually increase or decrease probability until a final probability is reached. This process is carried out by doctors in an intuitive but non-numerical way; the tool gives a precise number to the same process.

This tool has been developed as an app for a mobile phone as this has a number of advantages over alternative presentations. First, mobile phones are becoming much more prevalent throughout poorer countries and smartphones (which accommodate apps) are increasing their share of the market. Second, the information obtained can be retained easily on the phone for later use or uploaded to a webserver or emailed to another person. These functions could not be performed by, for example, a programmable calculator. The app has been developed for non-medical health workers and they used it in parts of the validation study. It should also be useful to inexperienced doctors who come across people with possible epilepsy.

**Figure 1:**
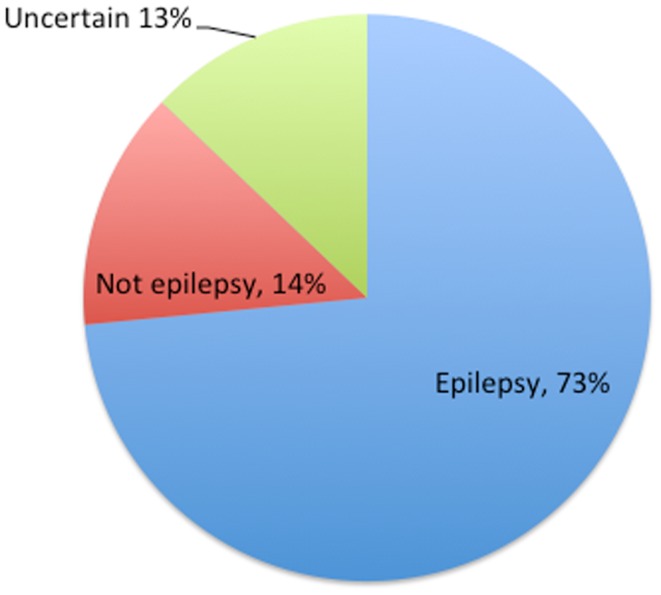
**Percentages of uncertain diagnoses as defined by mobile app in 132 patients**.

In practice, the app separates most people into probability scores suggestive of epilepsy or non-epilepsy. Just as in the gold-standard of history taking there are patients in whom the diagnosis is uncertain but these are relatively few (Figure 1).

Subsequently, health workers in Nepal have used the app in small numbers of patients with no false diagnoses.

The second diagnostic problem, whether epilepsy is primary or secondary is important because it leads directly to choice of medication. It too should be amenable to a Bayesian approach and work on this is in progress.

### Review

There are surprisingly few studies from anywhere in the world on using the telephone to review people with epilepsy. There is a randomized trial in progress comparing telephone with in-person review at a tertiary referral center in India (Mamta Singh, personal communication). Preliminary results from this have shown that there is no difference in breakthrough seizures between the groups but that the group reviewed by telephone had significantly fewer costs due to travel, accommodation, and lost wages. The same group has also shown that it is feasible to train nurses to review epilepsy patients in an Indian setting (17).

Telephone review is expedited by the high mobile phone ownership, which continues to increase in poorer countries. Communication technology infrastructure has improved in almost all countries of the world, however, poor. In particular, mobile phone usage has almost become universal and use of smartphones is increasing dramatically as these devices become cheaper (18). In another Indian study from 2011 showed that over half of patients with epilepsy who attended two rural epilepsy clinics were contactable by mobile phone 8 months later (12).

Text messaging using short messaging service (SMS) on mobile phones has been used as a way of continuing with epilepsy education in epilepsy patients under review (19). The authors of this study from Malaysia found that knowledge of epilepsy, medication adherence, and review attendance were all better in the group receiving SMS messages compared with a control group, which received conventional written information only.

## Future

The epilepsy treatment gap in the resource-poor parts of the world is unlikely to be narrowed substantially without using telemedicine approaches. These telemedicine approaches have at least started though they are still at an early stage and there is some evidence that they can contribute to epilepsy care. Once these methods have been refined then they can be deployed in larger scale trials but this will require significant investment in what is effectively an orphan area of medical research. Making this happen will require innovative ideas such as the global fund for epilepsy (20) to be realized. Then the benefits to millions of people in the resource-poor world with untreated epilepsy would be considerable.

## Conflict of Interest Statement

The author declares that the research was conducted in the absence of any commercial or financial relationships that could be construed as a potential conflict of interest.
